# Role of syringic acid in enhancing growth, photosynthesis, and antioxidant defense in lettuce exposed to arsenic stress

**DOI:** 10.1111/ppl.70051

**Published:** 2025-01-15

**Authors:** Melike Balci, Busra Arikan‐Abdulveli, Evren Yildiztugay, Ceyda Ozfidan‐Konakci

**Affiliations:** ^1^ Department of Biotechnology, Faculty of Science Selcuk University Selcuklu Konya TURKEY; ^2^ Department of Molecular Biology and Genetics, Faculty of Science Necmettin Erbakan University Meram Konya TURKEY

## Abstract

Heavy metal pollution, especially arsenic toxicity, significantly impairs plant growth and development. Phenolic acids, known for their antioxidant properties and involvement in stress signaling, are gaining increased attention as plant secondary metabolites with the potential to enhance plant resistance to these stressors. This study aimed to investigate the effects of different concentrations of syringic acid (SA1, 10 μM; SA2, 250 μM; SA3, 500 μM) on growth, photosynthetic parameters, and antioxidant activity in lettuce seedlings subjected to arsenic stress (As, 100 μM). Arsenic stress reduced growth by 56.7%, water content by 7.39%, and osmotic potential by 26.2% in lettuce leaves compared to control. Conversely, SA1 and SA2 treatments mitigated the adverse effects of arsenic on growth and preserved the water balance in plants. However, the SA3 treatment led to a decrease in growth by 18.9% and 39.5% in the SA3 and As+SA3 groups, respectively, indicating that high‐dose SA treatment adversely affected lettuce leaves under both control and stress conditions. Exogenous SA1 treatment significantly improved photosynthesis, whereas SA2 provided milder benefits and SA3 did not reduce the adverse effects of arsenic exposure. Arsenic stress increased H_2_O_2_ content by 47.3% and lipid peroxidation by 33.4% in lettuce seedlings. SA1 treatment effectively reduced oxidative stress by enhancing the activities of key antioxidant enzymes, such as superoxide dismutase (SOD) and peroxidase (POX). Moreover, SA1 was successful in maintaining the glutathione (GSH) pool, whereas SA2 primarily promoted ascorbate (AsA) regeneration. In conclusion, 10 μM of syringic acid (SA1) was identified as the optimal dose for reducing arsenic stress in lettuce by enhancing antioxidant activity and supporting growth. Overall, the findings underscore the potential of SA1 treatment in enhancing the resilience of lettuce to heavy metal toxicity.

## 1 INTRODUCTION

Arsenic (As) is an element recognized for its toxicity, and it poses a major threat to sustainable agriculture due to its growing concentrations in water, soil, and agricultural products as a result of climate change and industrialization (Sevak and Pushkar, 2024). While the environment contains both inorganic and organic As species, the forms of inorganic As that predominantly harm plants are arsenite As(III) and arsenate As(V) (Nazir et al., 2023). Due to its similarity to phosphate ions, As(V) is readily absorbed by plants and distributed to different regions of the plant through phosphate carriers. As(III) is formed by the reduction of As(V) in plant cells and has higher toxicity than arsenate, causing more harmful effects on plants (Attia and Alamer, 2024). Arsenic causes disruptions in many physiological and biochemical processes in plants, including decreased photosynthetic efficiency, inhibition of enzyme activities, lipid peroxidation, and impaired growth, resulting in abundant production of reactive oxygen species (ROS) and increased oxidative damage (Garg et al., 2024). Furthermore, research has indicated that induced ROSs are associated with the diminution of As from As(V) to As(III) (Piršelová et al., 2022). Plants have both enzymatic antioxidant defense systems such as superoxide dismutase, catalase, peroxidase, and non‐enzymatic antioxidant defense systems like ascorbate, glutathione, flavonoids, phenolics, and carotenoids to alleviate the adverse impacts of ROS (Samanta et al., 2022; Li et al., 2023).

Secondary metabolites, including phenolics, play a crucial role in functioning as signaling molecules and neutralizing ROS produced in plant cells under metal‐induced stress (Humbal and Pathak, 2023; Jan et al., 2023). Phenolic acids have attracted intense interest in scientific research because of their potential benefits (Kumar and Goel, 2019). They constitute the largest group of phenolic compounds, comprising hydroxycinnamic acids like caffeic and ferulic acids, as well as hydroxybenzoic acid derivatives such as syringic and gallic acids (Chen et al., 2020). Phenolic acids commonly contain a carboxyl group and a hydroxyl group attached to the aromatic ring, distinguishing them from other phenolics (Valanciene et al., 2020). The fact that they contain a hydroxyl group allows them to serve a critical function in eliminating free radicals in the plant defense system (Hilal et al., 2024). Additionally, phenolic acids exhibit antioxidant, antimicrobial, antiviral, and anticancer properties (Zhou et al., 2024b). Phenolics are an important bioactive group that prevents membrane lipid degradation by inhibiting lipid peroxidation (Tessema et al., 2023). Moreover, studies have shown that phenolic acids positively affect important processes like photosynthesis and protein synthesis in plants and may improve the stress resistance of plants under abiotic stress conditions (Sun et al., 2024).

Syringic acid (SA) is one of the important phenolic acids that stands out for these properties (Vo et al., 2020). Chemically recognized as 4‐hydroxy‐3,5‐dimethoxybenzoic acid, SA is a phenolic compound that is present naturally in plants such as olives, squash, grapes, acai berries wheat, maize and lettuce (John and Arockiasamy, 2021; Hameed et al., 2023; Somade et al., 2023; Yılmaz et al., 2024). SA is synthesized as a consequence of a series of enzymatic reactions via the shikimate pathway, which acts as a substrate in protein synthesis and the formation of phenolic compounds (Santos‐Sánchez et al., 2019). Synthesis begins with the amino acid phenylalanine and ends with the methylation of sinapic acid (Shimsa et al., 2024). SA may exhibit bioactive properties in the interaction between plants and bacteria (Bartel et al., 2023). It has been shown that SA enhances plant resistance to stress by regulating protein synthesis and stability, growth factors, and enzymatic activities in plant cells (Bandyopadhyay et al., 2024). In addition, according to a study conducted in animal cells, it was stated that SA may have a protective effect against heavy metal toxicity by preventing the formation of compounds that can cause oxidative damage (Akarsu et al., 2024). Furthermore, research indicates that SA decreases stress damages by reducing lipid peroxidation and ROS accumulation (John and Arockiasamy, 2020). These findings demonstrate that SA is a critical component that plays a vital role in supporting essential physiological and biological processes in plants. Ma et al., (2022) reported that SA application to tomato seedlings under lead (Pb) stress improved plant growth. However, research on exogenous applications of SA has been largely limited to animal cells. In this context, the available data on the potential tolerance that may be achieved through the application of this phenolic compound to plants is quite scarce and further research is needed. Providing data on reducing or eliminating the impacts of heavy metal pollution on plants, particularly those increasing due to climate change, is of critical importance. Therefore, we decided to conduct this study to understand the potential of SA to increase stress tolerance in plants.

Lettuce (*Lactuca sativa* L.) is a commonly grown leafy vegetable recognized for its nutritional and economic significance (Farooq et al., 2023). It is rich not only in minerals, fibers, and vitamins but also in phenolic acids and flavonoids (Akca et al., 2024). Research has revealed metal accumulation in lettuce leaves cultivated in soil polluted with heavy metals (Yuce et al., 2024). This situation may pose severe risks to both plant health and human health (Tang et al., 2023). In this context, understanding the effects of arsenic stress on lettuce is crucial for effectively activating the defense mechanisms of plants against this stress. Although there are studies investigating the impacts of As stress on plants and external applications of phenolic compounds to mitigate the impress of stress, no research exists investigating the impacts of SA applications on lettuce seedlings under As stress. Therefore, the purpose of this study was to examine the impact mechanisms of SA application on photosynthetic parameters and antioxidant enzyme/isozyme activities on lettuce leaves under As stress. Furthermore, in this research, the effects on relative water content, osmoregulation, ROS content and lipid peroxidation levels in SA‐treated lettuce leaves were investigated.

## 2 MATERIALS AND METHODS

### 2.1 Plant specimen and study design

Lettuce (*Lactuca sativa* L. cv. *Yedikule*) seedlings were grown in a Hoagland solution under controlled hydroponic conditions. 100 μM sodium arsenate (Na_2_HAsO_4_•7H_2_O) was applied for arsenic application, following the protocols established in previous research by (da‐Silva et al., 2018; Alatawi et al., 2023). The syringic acid (S6881, Sigma‐Aldrich) doses we used in our study were determined as a result of our previous preliminary trials and were selected as SA1, 10 μM; SA2, 250 μM and SA3, 500 μM. Arsenic and SA treatments were applied to 21‐day‐old healthy seedlings. Plants were harvested after three days of stress exposure and stored at ‐80°C for physiological and biochemical analysis.

### 2.2 Measurement of physiological parameters

Six plants were used for the control group and each treatment group. Fresh weights (FW) of the leaves were measured with a digital scale. After the samples were oven dried at 70°C, dry weights (DW) were measured with a digital scale. The relative growth rate (RGR) values were calculated according to the following formula by Hunt et al., (2002).

$$\mathrm{RGR}=\left[\ln\;\left({\mathrm{DW}}_{2}\right)–\ln\;\left({\mathrm{DW}}_{1}\right)\right]/\left(t_{2}–t_{1}\right)$$

where DW_1_ = dry weight (g) at t1; DW_2_ = dry weight (g) at t_2_, t_1_; initial harvest and t_2_; final harvest.

After one week of the treatment period, six leaves were harvested, and their fresh weight (FW) was determined. The leaves were floated on de‐ionized water for 6 h and the turgid tissue was blotted dry prior to determining turgid weight (TW). Dry weight (DW) was determined after oven drying at 70°C. The leaf relative water content (RWC) was calculated by the following formula: Maghsoudi et al., (2020).

$$\mathrm{RWC}\;\left(\%\right)=\left[\left(\mathrm{FW}-\mathrm{DW}\right)/\left(\mathrm{TW}-\mathrm{DW}\right)\right]\;x\;100$$

Changes in osmotic potentials of lettuce leaves of each group were measured in mmol kg^−1^ with a Wescor 5600 brand osmometer. Then, the results were converted to MPa by multiplying with the coefficient 2.408x10^−3^ according to Santa‐Cruz et al., (2002).

Proline (Pro) levels were determined in 0.5 g of fresh specimens, employing the protocol outlined by Chandrakar et al., (2016).

### 2.3 Measurement of chlorophyll *a* fluorescence parameters and OJIP test

A portable fluorometer (FMS‐2, Hansatech) was used to determine the maximal quantum yield of PSII photochemistry (F_v_/F_m_). The descriptions for the estimated parameters are included in Supplementary Table S1. The radar plots depict the mean parameter values of different treatments in leaves (Figure 3D).

Carbon assimilation rate (A_c_), stomatal conductance (g_s_), intercellular CO_2_ concentration (C_i_), and transpiration rate (*E*) were measured with a portable gas exchange system (LCpro+; ADC). Gas exchange parameters were measured in six replicates by selecting leaves of similar size from each treatment group at the end of the seven‐day experimental setup.

### 2.4 Analysis of oxidative stress markers H₂O₂ and TBARS

The leaf H₂O₂ concentration was assessed according to the procedure explained by Velikova et al., (2000). The H_2_O_2_ content of 0.1 g leaf tissue harvested from the treatment groups for each biological replicate, with the attention to uniformity, was measured using the eFOX reagent (250 μM ferro ammonium sulfate, 100 μM xylenol orange, 100 μM sorbitol and 1% ethanol (v/v). The sample and eFOX reagent mixture was incubated for 30 minutes at room temperature. Then, absorbance measurements were performed at 550 and 800 nm. H_2_O_2_ concentrations in the samples were calculated according to the standard curve prepared according to known H_2_O_2_ amounts.

Lipid peroxidation (Thiobarbituric acid reactive substances (TBARS) content) of 0.1 g leaf tissue harvested from treatment groups for each biological replicate, with the attention to uniformity, was determined by making minor modifications to the method of Rao and Sresty (2000). The TBARS concentration of leaf samples was calculated from the absorbance at 532 nm, and measurements were corrected for nonspecific turbidity by subtracting the absorbance at 600 nm. The concentration of TBARS was calculated using an extinction coefficient of 155 mM^−1^ cm^−1^.

### 2.5 Identification of enzymatic and non‐enzymatic antioxidant enzyme compositions

Fresh, fully expanded leaves from the treatment groups were collected with attention to uniformity. 0.5 g leaf tissue was homogenized in Tris–HCl buffer and then centrifuged at 14 000 g for 30 minutes. The supernatants obtained were collected to measure total protein content using the method outlined by Bradford (1976). In this study, both kinetic and electrophoretic analysis were performed to comprehensively evaluate enzyme activity. While kinetic analysis provided quantitative measurements of total enzyme activity, gel analysis allowed visualization and identification of specific isoforms, offering insights into their differential regulation under experimental conditions.

For superoxide dismutase (SOD, EC 1.15.1.1) isozyme activity, samples were subjected to non‐denaturing polyacrylamide gel electrophoresis (PAGE) as described by Laemmli (1970). Extracts were run at a constant current of 120 mA on a 12% separating gel and a 5% stacking gel in a Biorad Mini‐Protean electrophoresis system. Total SOD activity was determined using riboflavin and nitroblue tetrazolium (NBT) staining, as described by Beauchamp and Fridovich (1971). After incubating the gels in the staining solution for 30 minutes, SOD isozyme bands were visualized using a gel imaging system, and their density was analyzed using the Bio‐1D software. Catalase (CAT, EC 1.11.1.6) isozymes were detected according to Woodbury et al., (1971). The reduction in H₂O₂ concentration was monitored spectrophotometrically by measuring the decrease in absorbance at 240 nm. The reaction mixture in a 1 ml quartz cuvette consisted of 0.1 mM EDTA, 50 mM sodium phosphate buffer (pH 7), deionized water, and 0.3% H₂O₂. Absorbance changes were recorded over 180 seconds. CAT activity was expressed as μmol of H₂O₂ consumed per minute. Total CAT activity was estimated according to the method of Bergmeyer (1970). Samples with equal protein content were run on a 7.5% native gel, incubated in 0.01% H₂O₂ for 5 minutes, and washed with deionized water. The gels were then stained for 20 minutes with a solution containing 1% FeCl₃ and 1% K₃Fe(CN)₆. To detect specific CAT isozymes, 3‐amino‐1,2,4‐triazole (3‐AT) was added to the H₂O₂ solution. Stained gels exhibiting bands were rinsed with deionized water. The isozymes and enzyme activity of peroxidase (POX, EC 1.11.1.7) were based on the method described by Seevers et al., (1971) and Herzog and Fahimi (1973), respectively. The amount of oxidized DAB (3′‐3′‐diaminobenzidine tetrahydrochloride) in the presence of H₂O₂ was measured at 465 nm against a blank over 3 minutes. The reaction mixture in the polystyrene cuvette consisted of DAB solution, 0.6% H₂O₂, deionized water, and enzyme extract. The reaction was initiated by adding H₂O₂, and the increase in absorbance was recorded. For POX isozyme separation, equal protein samples were resolved on a 10% native acrylamide gel at a constant current of 120 mA. Post‐electrophoresis, gels were incubated for 30 minutes in the dark in 200 mM sodium acetate buffer (pH 5.0) containing benzidine and hydrogen peroxide. NADPH oxidase (NOX, EC 1.6.3.1) isozymes were identified as described by Sagi and Fluhr (2001). NOX activity was measured according to Jiang and Zhang (2002). The enzyme/isozyme activities of glutathione S‐transferase (GST, EC 2.5.1.18) were determined (Ricci et al., 1984; Hossain et al., 2006). The reaction mixture contained 100 mM Tris–HCl buffer (pH 6.5), 1.5 mM GSH, and 1 mM 1‐chloro‐2,4‐dinitrobenzene (CDNB). The reaction was initiated by adding 20 μl of the sample to 980 μl of the reaction mixture. The increase in absorbance at 340 nm was monitored for 3 minutes. The enzyme activity was calculated using an extinction coefficient of 9.6 mM^−1^ cm^−1^. GST isozymes were separated using 10% native PAGE electrophoresis. Following electrophoresis, the gels were incubated in 0.1 M K‐P buffer (pH 6.5). The gels were then transferred to a solution containing 4.5 mM GSH, 1 mM CDNB, and 1 mM NBT in 0.1 M K‐P buffer (pH 6.5). GST bands were visualized using a staining solution containing 3 mM PMS in 0.1 M Tris–HCl buffer (pH 9.6). The isozymes and enzyme activity of glutathione peroxidase (GPX, EC 1.11.1.9) activity were based on the method described by Lin et al., (2002) and Elia et al., (2003) respectively. The reaction mixture consisted of 100 mM sodium phosphate buffer (pH 7.5), 1 mM EDTA, 1 mM sodium azide, 0.12 mM NADPH, 2 mM GSH, 1 U GR, and 0.6 mM H₂O₂. The reaction was initiated by adding 20 μl of the sample to 980 μl of the reaction mixture. The oxidation of NADPH was monitored spectrophotometrically at 340 nm for 1 minute, and the activity was calculated using an extinction coefficient of 6.62 mM^−1^ cm^−1^. GPX isozymes were separated by electrophoresis, gels were transferred to 50 mM Tris–HCl buffer (pH 7.9) for 20 minutes, then to a solution containing 13 mM GSH and 0.004% H₂O₂ for gentle shaking. Isozymes were visualized by incubating the gels in 1.2 mM MTT and 1.6 mM PMS in distilled water in the dark for 10 minutes.

Electrophoretic ascorbate peroxidase (APX, EC 1.11.1.11) separation was performed according to Mittler and Zilinskas (1993). APX enzyme activity was measured according to Nakano and Asada (1981). The decrease in absorbance at 290 nm, corresponding to the oxidation of ascorbate, was recorded. The reaction mixture contained 50 mM sodium phosphate buffer (pH 7.0), 0.5 mM ascorbate, 0.1 mM EDTA‐Na₂, and 1.2 mM H₂O₂. The oxidation of ascorbate was initiated by adding enzyme extract, and the decrease in absorbance was followed for 180 seconds. One unit of APX activity was defined as the amount of enzyme required to oxidize 1 μmol of ascorbate per minute per ml. Glutathione reductase (GR, EC 1.6.4.2) activity was measured according to Foyer and Halliwell (1976). The decrease in NADPH absorbance due to the reduction of GSSG was monitored in a quartz cuvette for 180 seconds. The activity was calculated using an extinction coefficient of 6.2 mM^−1^ cm^−1^. Specific enzyme activity was expressed as the amount of GSSG reduced per minute per ml. Isozymes compositions of GR were determined by native PAGE analysis (Hou et al., 2004). After electrophoresis, the gels were incubated for 20 minutes in a solution containing 4 mM GSSG, 1.5 mM NADPH, and 2 mM 5,5′‐dithiobis (2‐nitrobenzoic acid; DTNB) in 10 mM Tris–HCl buffer (pH 7.9). The gels were rinsed briefly with 50 mM Tris–HCl buffer (pH 7.9) and stained negatively with 1.2 mM MTT, 1 mM 2,6‐dichloroindophenol, and 1.6 mM N‐methylphenazonium methyl sulfate for 5 minutes to visualize the bands.

Monodehydroascorbate reductase (MDHAR; EC 1.6.5.4) activity was assayed by the method of Miyake and Asada (1992). The reaction mixture contained 50 mM Tris–HCl buffer (pH 7.5), 0.2 mM NADPH, 2.5 mM AsA, and 0.5 units of ascorbate oxidase (AO). The reaction was initiated by adding AO, and the decrease in absorbance at 340 nm was monitored for 1 minute. Enzyme activity was calculated using an extinction coefficient of 6.2 mM^−1^ cm^−1^. Dehydroascorbate reductase (DHAR; EC 1.8.5.1) activity was measured according to Dalton et al., (1986). The reaction mixture contained 50 mM potassium phosphate buffer (pH 7.0), 2.5 mM GSH, and 0.1 mM dehydroascorbate (DHA). The activity was initiated by adding the sample solution to the reaction mixture. The decrease in absorbance at 265 nm was monitored for 180 seconds, and the activity was calculated using the extinction coefficient of 14 mM^−1^ cm^−1^. Total and reduced ascorbate (AsA) contents were done according to the method of Dutilleul et al., (2003) with modifications. The oxidized form of ascorbate (DHA, dehydroascorbate) was measured using the formula DHA = Total AsA‐Reduced AsA. The glutathione (GSH) was assayed according to Paradiso et al., (2008). Oxidized glutathione (GSSG) was determined after the removal of GSH by 2‐vinylpyridine derivatization. GSH redox state (%) was determined by calculating the ratio of GSH to total glutathione (GSH + GSSG) according to Shi et al., (2013).

Gels stained for SOD, CAT, POX, APX, GR, GST, GPX, and NOX activities were photographed with the Gel Doc XR+ System and then analyzed with Image Lab software v4.0.1 (Bio‐Rad). Known standard amounts of enzymes (0.5 units of SOD and 0.2 units of CAT and POX) were loaded onto gels. For each isozyme set/group, the average values were significantly different at *p* < 0.05 using the Tukey's post‐test.

### 2.6 Statistical analysis

Each measurement was performed in technical triplicates and conducted on six separate biological samples. A one‐way ANOVA was used to perform the statistical analysis. SPSS version 20.0 software was utilized to conduct the statistical evaluation of the data. OriginPro2022 software was used to construct radar charts. A significance level of p < 0.05 was employed to evaluate statistical significance.

## 3 RESULTS

### 3.1 Growth characteristics of syringic acid‐treated lettuce seedlings under As stress

**FIGURE 1:**
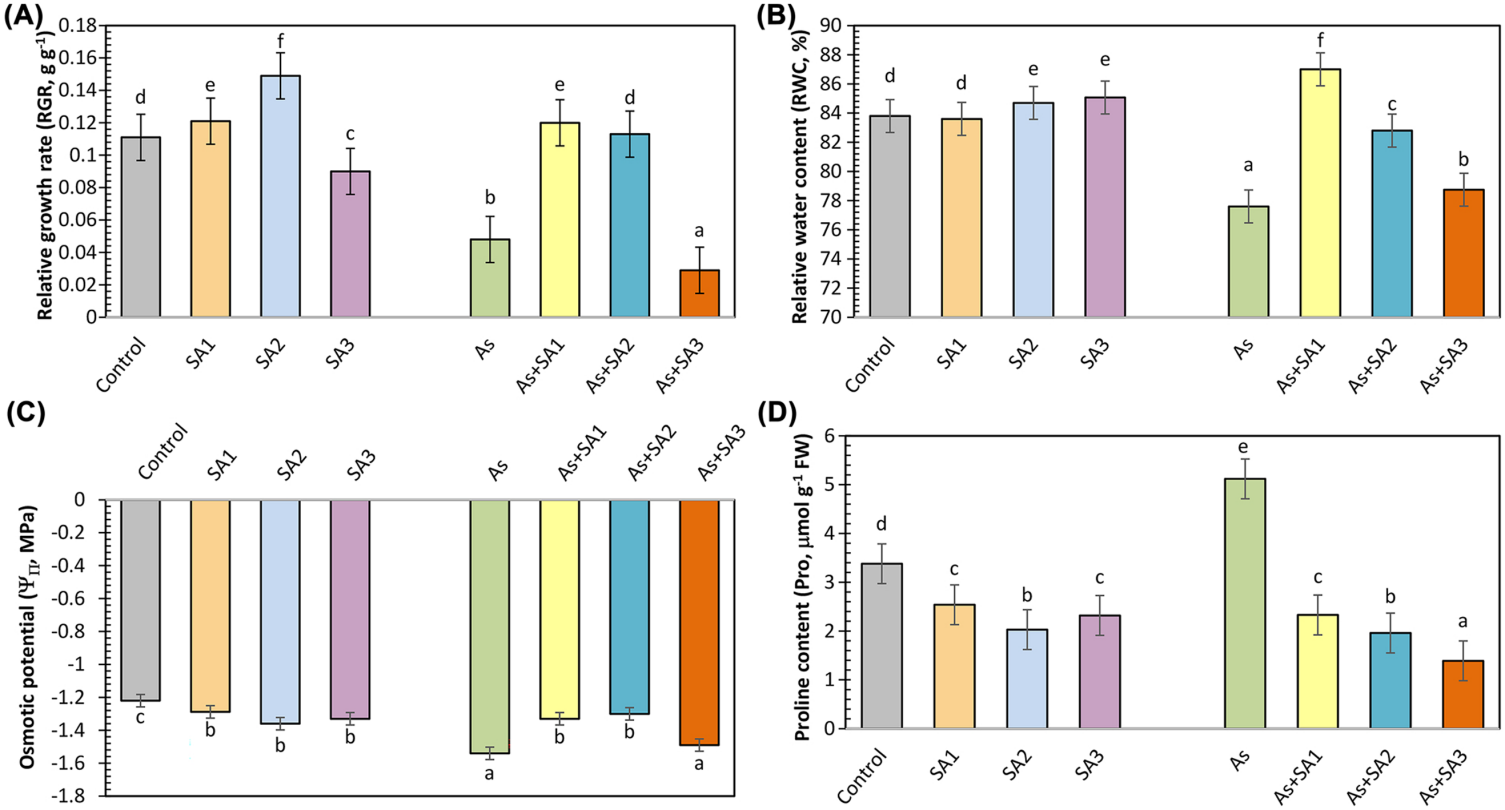
Effect of syringic acid treatment on physiological parameters in lettuce leaves under arsenic stress. The relative growth rate (RGR, A), relative water content (RWC, B), osmotic potential (Ψπ, C) and proline content (Pro, D) in syringic acid (SA1, 10 μM; SA2, 250 μM; SA3, 500 μM) treated lettuce leaves under arsenic stress (As, 100 μM) (*n* = 6). All data obtained were subjected to a one‐way analysis of variance (ANOVA). Differences were considered to be significant at *p* < 0.05.

In our study, SA1 and SA2 treatment groups enhanced the relative RGR by 9% and 34%, sequentially, relative to the control, while SA3 caused a decrease of 18.9% in lettuce plants (Figure 1A). Under arsenic stress, RGR decreased dramatically by 56.8% in comparison with the control. However, SA1 and SA2 treatments with stress alleviated some of the growth reduction, with RGR values improving to levels comparable to the control. However, As+SA3 treatment led to an even further reduction in RGR. The RWC did not show significant changes in SA1 treatment relative to control, whereas SA2 and SA3 showed slight increases in their treatments (Figure 1B). Under arsenic stress, RWC reduced by 7.39% against the control. As+SA1 and As+SA2 groups enhanced RWC to a higher level than the control, with an increase of 12.1% and 6.7%, respectively. Osmotic potential (Ψπ) decreased under As stress, showing a 26.2% decrease relative to the control (Figure 1C). SA treatments generally reduced Ψπ compared to the control, with SA1, SA2 and SA3 showing a reduction of 5.6%,11.6% and 9%, respectively. Co‐treatment with SA1 and SA2 mitigated the decrease caused by As, restoring Ψπ to levels closer to the control (Figure 1C). The As+SA3 group showed a partial recovery but still had a lower Ψπ as opposed to the control. Pro content increased significantly under As stress, rising by 51.4% compared to the control (Figure 1D). SA treatments reduced Pro content, with SA1 showing a decrease of 24.8%, SA2 showing a 39.9% decrease, and SA3 showing a 31.3% decrease relative to the control. SA1, SA2 and SA3 treatments together with stress effectively reduced Pro content by 54.5%, 61.7%, and 72.8%, respectively, indicating a reduction in stress levels.

### 3.2 Effects of syringic acid treatments on gas exchange and photosynthesis parameters of lettuce leaves under As stress

**FIGURE 2:**
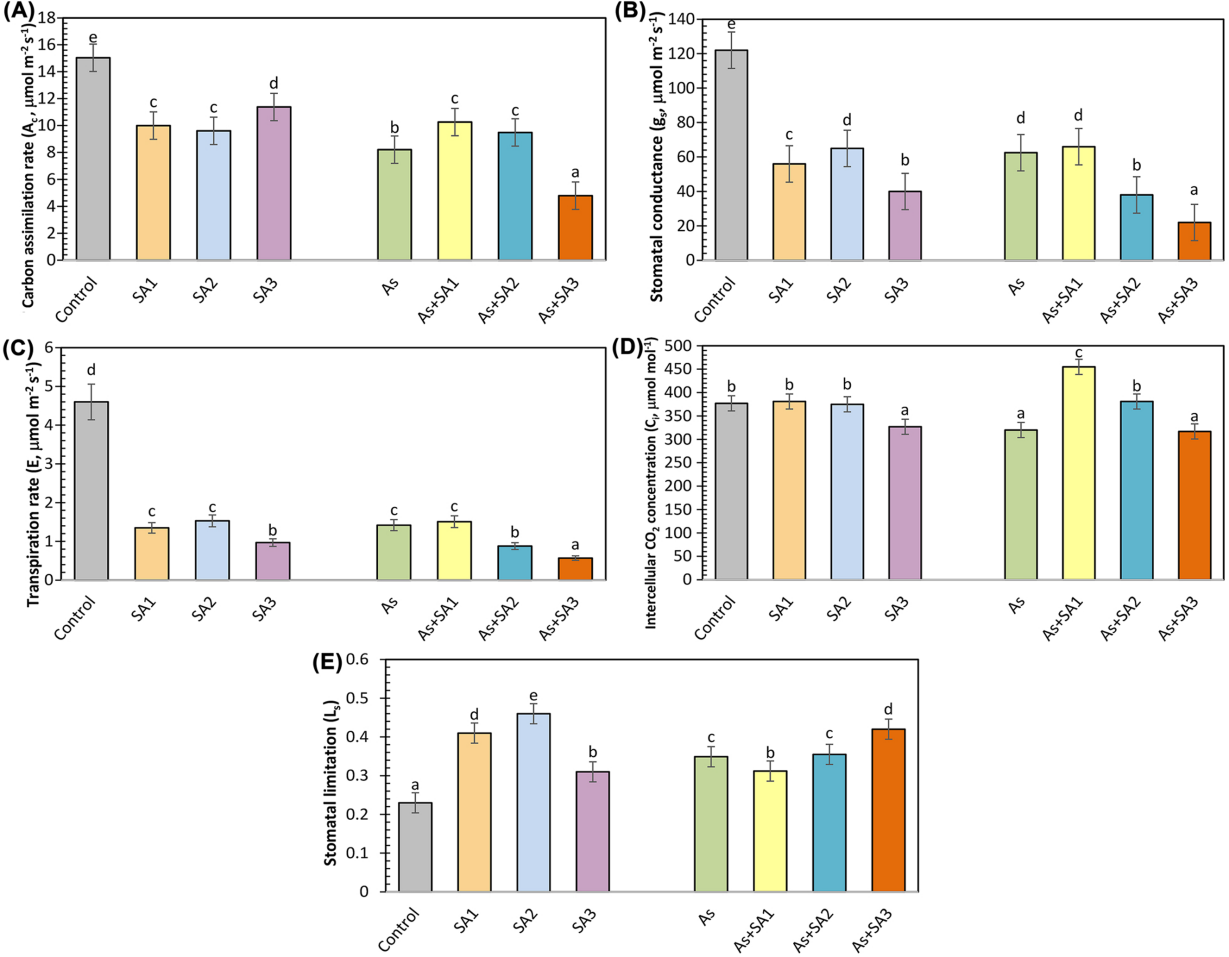
Effect of syringic acid treatment on gas exchange parameters in lettuce leaves under arsenic stress. Carbon assimilation rate (A_c_, A), stomatal conductance (g_s_, B), transpiration rate (E, C), intercellular CO_2_ concentrations (C_i_, D) and stomatal limitation rate (L_s_, E) in syringic acid (SA1, 10 μM; SA2, 250 μM; SA3, 500 μM) treated lettuce leaves under arsenic stress (As, 100 μM; *n* = 5). All data obtained were subjected to a one‐way analysis of variance (ANOVA). Differences were considered to be significant at p < 0.05.

The A_c_ decreased by 33.5%, 36.1% and 24.3% in SA1, SA2 and SA3 groups, respectively, when compared relative to the control group (Figure 2A). Under As stress, the A_c_ value was reduced by 45.4% in comparison with the control. Relative to As stress, SA treatments improved by 24.9% and 15.5% in As+SA1 and As+SA2 groups, respectively, while it mitigated by 41.6% in As+SA3 group. g_s_ was notably decreased with all SA treatments (Figure 2B). SA1, SA2, and SA3 treatments led to reductions of 54%, 46.7%, and 67.2%, respectively, against the control. Arsenic stress led to a 48.7% decrease in g_s_ compared to the control. SA treatment with As stress had little effect on g_s_, with only the As+SA1 group showing a slight increase relative to the arsenic group, but this difference lacked statistical significance. Transpiration rate (*E*) was significantly decreased by all SA treatments, with the SA1, SA2, and SA3 groups showing reductions of 70.6%, 66.7%, and 78.9%, respectively, as opposed to the control (Figure 2C). Arsenic stress decreased *E* by 69.1% in comparison with the control. Relative to the stress group, SA treatment in plants did not demonstrate an important change in *E* in the As+SA1 group, while a reduction of 38% in the As+SA2 group and 59.8% in the As+SA3 group was detected. C_i_ showed no significant difference in the SA1 and SA2 treatments compared to normal conditions, while a 13.2% decrease was observed in the SA3 treatment (Figure 2D). Arsenic stress led to a 15.1% decrease in C_i_ relative to the control. However, under As stress, the SA1 treatment increased by 42.2%, the SA2 treatment by 19%, while no significant change was recorded in the SA3 treatment. As opposed to the control conditions, the stomatal limitation value (L_s_) increased by 78.2% and 34.7% in SA1 and SA3 groups, respectively, while a 2‐fold increase was observed in SA2 group (Figure 2E). In addition, L_s_ level increased by 51.7% under As stress relative to normal conditions. However, in the As+SA1 group, a 10.6% decrease was observed. No major difference was noted in the As+SA2 group, and a 20.3% increase was detected in the As+SA3 group.

**FIGURE 3:**
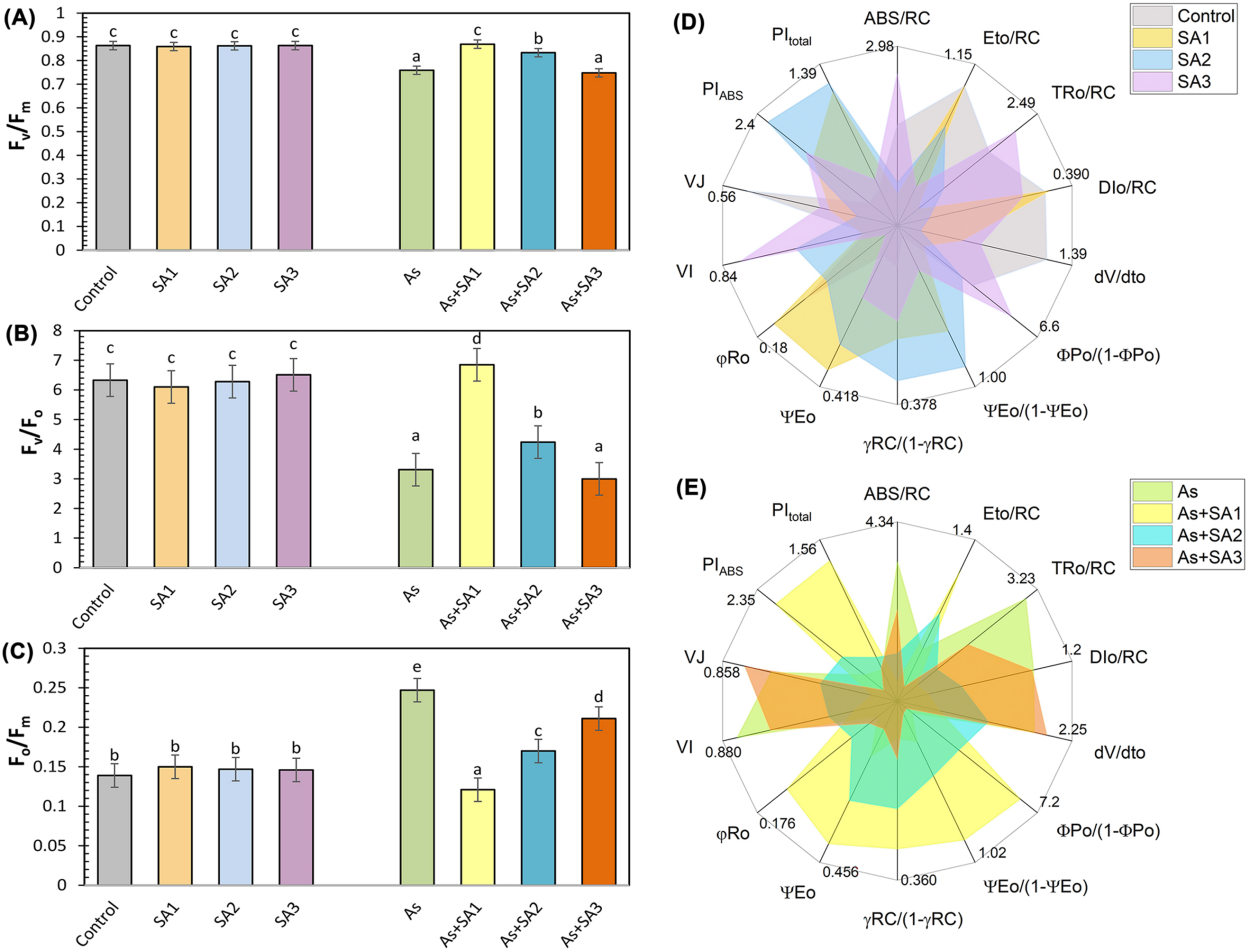
Effect of syringic acid treatment on photosynthetic parameters in lettuce leaves under arsenic stress. The maximal quantum yield of PSII photochemistry (F_v_/F_m_, A), potential photochemical efficiency (F_v_/F_o_, C), physiological state of the photosynthetic apparatus (F_o_/F_m_, C) and OJIP transient radar plots (D‐E) in syringic acid (SA1, 10 μM; SA2, 250 μM; SA3, 500 μM) treated lettuce leaves under arsenic stress (As, 100 μM; n = 5). All data obtained were subjected to a one‐way analysis of variance (ANOVA). Differences were considered to be significant at p < 0.05.

Chlorophyll fluorescence parameters, including maximum quantum yield of photosystem II (F_v_/F_m_) and quantum yield of PSII photochemistry (F_v_/F_o_), were largely unaffected by SA treatments (Figure 3A, B). Under As stress, F_v_/F_m_ and F_v_/F_o_ were significantly reduced by 12.1% and 47.7%, sequentially, in comparison with the control. SA treatment with stress alleviated the As‐induced decline, particularly in the As+SA1 group, where F_v_/F_m_ increased by 14.5% compared to the As group. The minimum quantum yield of PSII (F_o_/F_m_) ratio was elevated under As stress (77.7%), but was restored by SA treatment, with the As+SA1 group showing a 51% reduction compared to arsenic alone (Figure 3C). The radar plots illustrating the JIP test parameters from our study is presented in Figure 3D‐E. In lettuce seedlings exposed to As stress, an increase in ABS/RC, TRo/RC, DIo/RC, dV/dto, VI, and VJ values was observed against the control, while a reduction was noted in Eto/RC, ΦPo/(1‐ΦPo), ΨEo/(1‐ΨEo), γRC/(1‐γRC), ΨEo, φRo, PI_ABS_, and PI_total_ values. SA treatment reversed most of these parameters. Especially in performance indices (PI_ABS_ and PI_total_), it was determined that SA alone and SA treatment with stress had a positive effect on these parameters (except As+SA3).

### 3.3 H_2_O_2_ accumulation and lipid peroxidation levels of syringic acid treated lettuce plants under As stress

**FIGURE 4:**
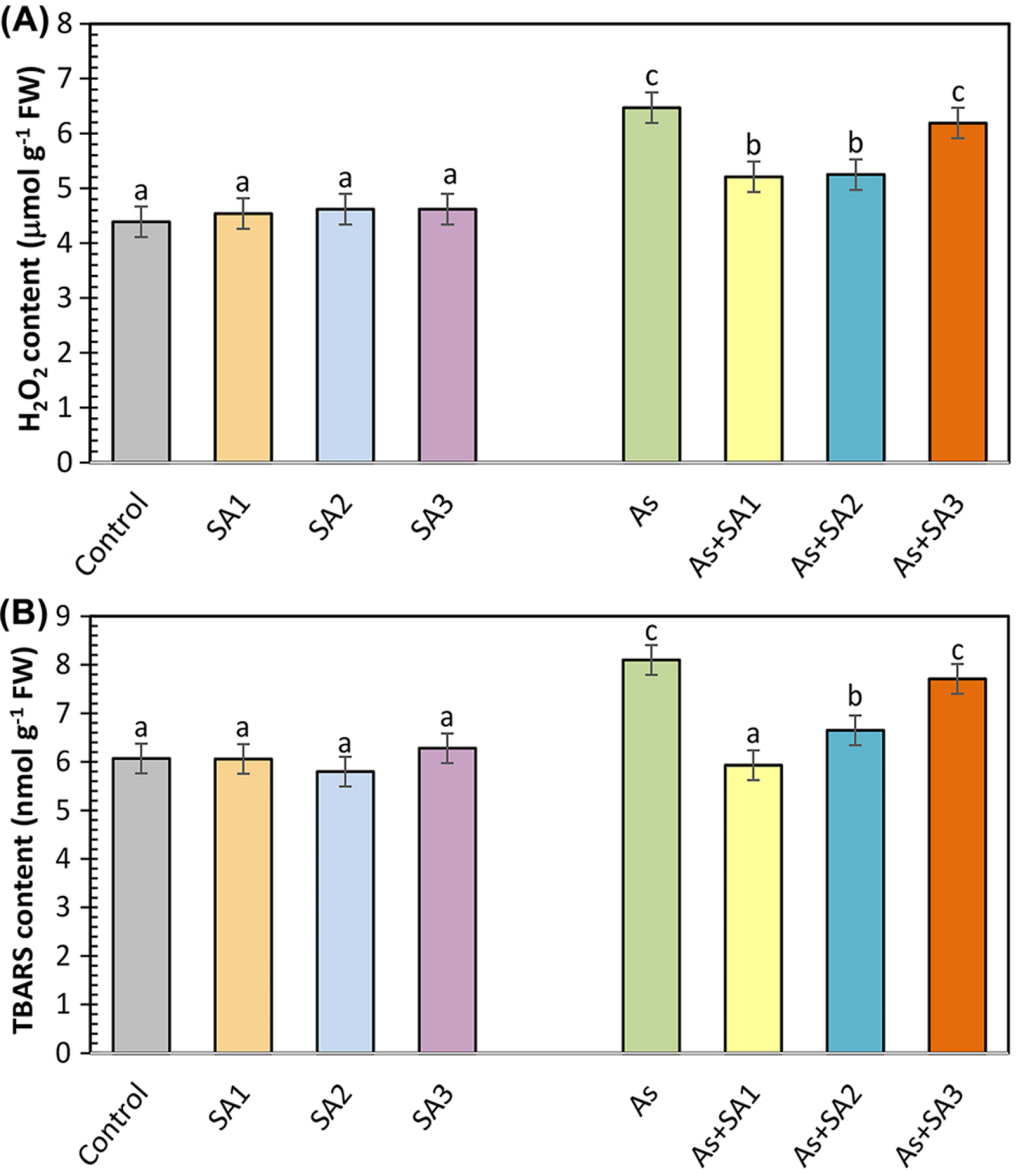
Impact of syringic acid treatment on oxidative stress indicators in lettuce leaves under arsenic stress. Hydrogen peroxide (H_2_O_2_, A) and lipid peroxidation (TBARS, B) contents in syringic acid (SA1, 10 μM; SA2, 250 μM; SA3, 500 μM) treated lettuce leaves under arsenic stress (As, 100 μM; n = 6). All data obtained were subjected to a one‐way analysis of variance (ANOVA). Differences were considered to be significant at p < 0.05.

H_2_O_2_ content was unaffected by SA treatments under non‐stress conditions but increased by 47.3% under As stress in comparison with the control (Figure 4A). SA treatment with stress reduced H_2_O_2_ accumulation especially in As+SA1 and As+SA2 groups, and a decrease of 19.5% and 18.8% was observed compared to the As treated group, respectively. Lipid peroxidation, measured as TBARS content, showed minimal variation with SA treatments under non‐stress conditions (Figure 4B). However, arsenic stress increased TBARS by 33.4% relative to the control. SA treatment together with stress reduced TBARS content by 26.7% and 17.9% in As+SA1 and As+SA2 groups, respectively, compared to the As group. No notable change was seen in the As+SA3 group.

### 3.4 Effects of syringic acid treatments on enzymatic and non‐enzymatic antioxidant enzyme profiles of lettuce leaves under As stress

**FIGURE 5:**
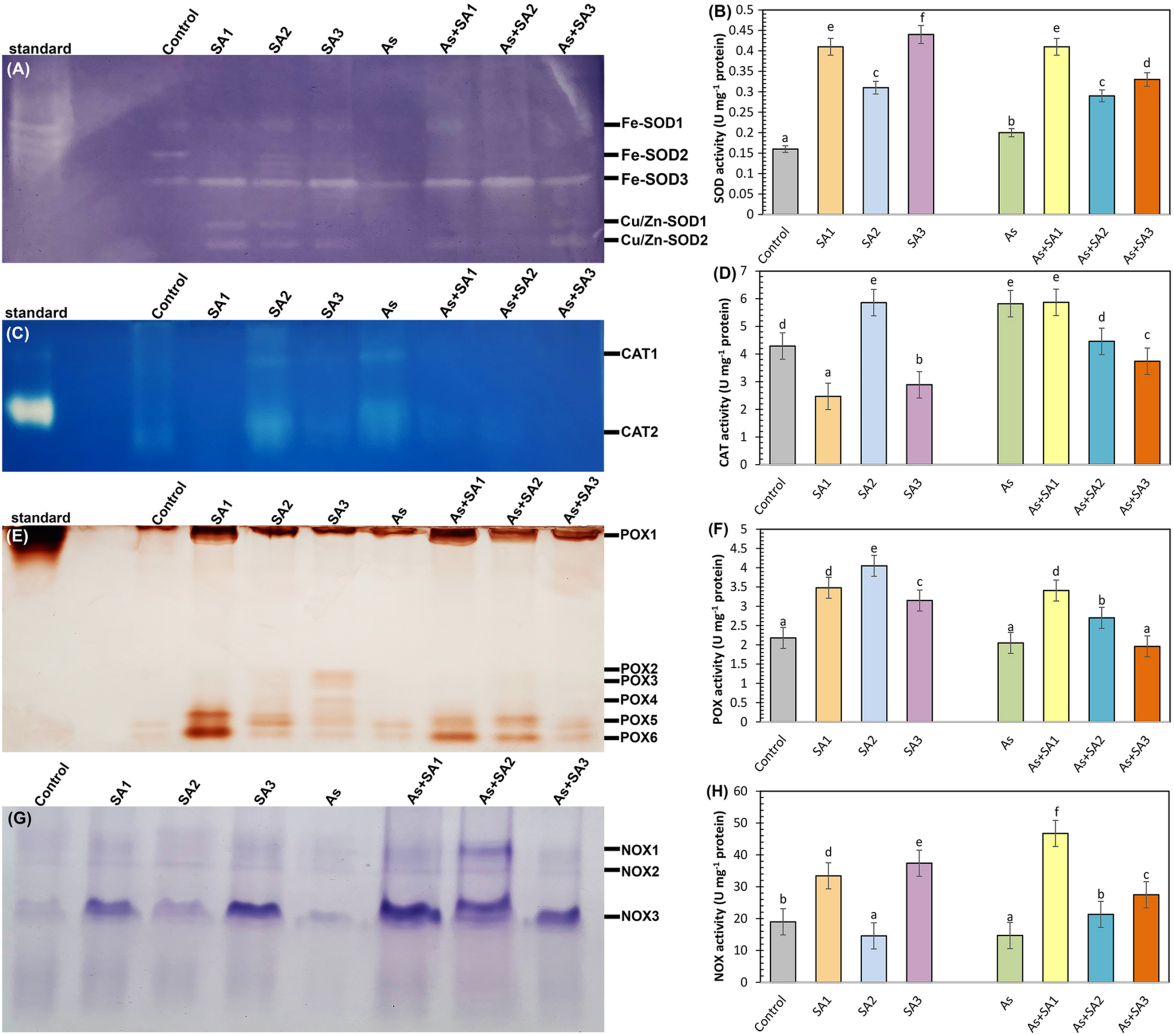
Effect of syringic acid treatment on antioxidant enzyme/isoenzyme profiles and activities in lettuce leaves under arsenic stress. Relative band intensity of different types of superoxide dismutase isoenzyme profiles (SOD, A) and SOD activity (B), relative band intensity of different types of catalase isoenzyme profiles (CAT, C) and CAT activity (D), relative band intensity of different types of peroxidase isoenzyme profiles (POX, E) and POX activity (F), relative band intensity of NADPH oxidase isoenzyme profiles (NOX, G) and NOX activity (H) in syringic acid (SA1, 10 μM; SA2, 250 μM; SA3, 500 μM) treated lettuce leaves under arsenic stress (As, 100 μM; n = 6).

Native page analysis of SOD revealed five isoforms, including three Fe‐SOD and two Cu/Zn‐SOD bands in lettuce leaves (Figure 5A). SA1 and SA3 treatments significantly increased total SOD activity by 2.5‐fold and 2.7‐fold, and SA2 treatment by 93.7%, compared with the control (Figure 5B). Under As stress, SOD activity was increased by 25%. SA treatment with stress further increased SOD activity. The highest enhancement was demonstrated 2‐fold in the As+SA1 group. Native‐PAGE analysis identified two CAT isoenzyme bands in lettuce plants (Figure 5C). CAT activity decreased by 42.4% and 32.6% with SA1 and SA3 treatments, respectively, as opposed to the control, while an increase of 36.5% was recorded with the SA2 treatment. (Figure 5D). Arsenic stress led to a 35.6% improvement in CAT activity. On the other hand, although there was no statistical distinction in CAT activity in the As+SA1 group, a decrease of 23.3% and 35.7% was observed in the As+SA2 and As+SA3 groups, respectively. As a result of the examined gel images, six POX isoenzyme bands were observed (Figure 5E). There was no visible change in POX activity in response to arsenic stress compared to the control (Figure 5F). However, SA treatment increased POX activity under non‐stressed and stressed conditions (except for the As+SA3 group). Three NOX isoenzyme bands were identified during the study (Figure 5G). NOX activity increased substantially with SA1 and SA3 treatments by 76% and 97% against the control (Figure 5H). Arsenic stress reduced NOX activity by 22.5%. All SA treatments increased NOX activity compared to the stress group. The largest growth was detected in the As+SA1 group (3,1‐ fold).

**FIGURE 6:**
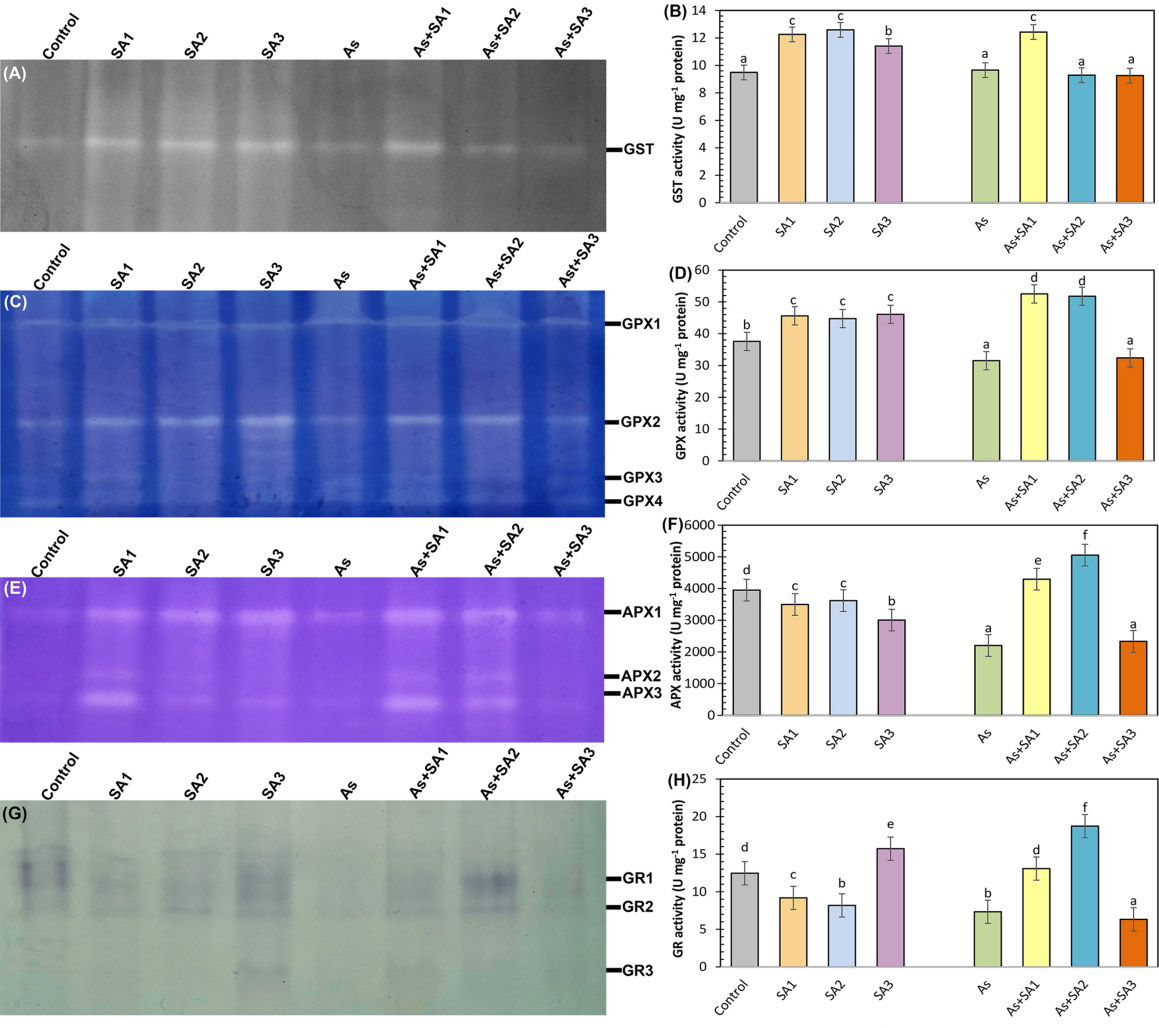
Impact of syringic acid treatment on antioxidant enzyme/isoenzyme profiles and activities in lettuce leaves under arsenic stress. Relative band intensity of different types of glutathione S‐transferase isoenzyme profiles (GST, A) and GST activity (B), relative band intensity of different types of glutathione peroxidase isoenzyme profiles (GPX, C) and GPX activity (D), relative band intensity of ascorbate peroxidase isoenzyme profiles (APX, E) and APX activity (F), relative band intensity of glutathione reductase isoenzyme profiles (GR, G) and GR activity (H) in syringic acid (SA1, 10 μM; SA2, 250 μM; SA3, 500 μM) treated lettuce leaves under arsenic stress (As, 100 μM; n = 6).

We presented in Figure 6A that a GST isoenzyme was detected in lettuce leaves. GST activity increased by 29.2%, 32.6% and 20.2% in SA1, SA2 and SA3 groups, respectively, compared to control (Figure 6B). Arsenic stress did not demonstrate any considerable change in GST activity relative to control plants. Additionally, SA‐treated plants did not show any significant difference on GST activity in the presence of stress, except for the As+SA1 group. Regarding the GPX enzyme, four isozyme bands were identified in lettuce leaves (Figure 6C). Only SA treatments induced GPX activity (Figure 6D). While arsenic stress suppressed GPX activity against the control, SA treatments together with stress alleviated this situation and induced GPX activity (except As+SA3 group). Native‐PAGE analysis identified three APX isoenzyme bands in lettuce plants (Figure 6E). APX activity decreased by 11.4% in the SA1 treatment, 8.4% in SA2, and 23.8% in SA3 as opposed to the control (Figure 6F). Arsenic stress significantly decreased APX activity by 44.1%. However, SA treatment with stress significantly increased APX activity by 94.8% in the As+SA1 group and 2.2‐fold in the As+SA2 group. Additionally, the As+SA3 group did not alter the negative impact of arsenic stress. Three GR isoenzyme bands were observed in the gel images (Figure 6G). GR activity decreased by 26.2% and 34.3% in SA1 and SA2 groups, respectively, compared to control, while it increased by 26.1% in SA3 group (Figure 6H). Arsenic stress causes a 41% reduction in GR activity, which was restored in the As+SA1 and As+SA2 groups by 78.2% and 2.5‐ fold, in that order, relative to the As group. However, a 13.7% decrease was determined in the As+SA3 group.

**FIGURE 7:**
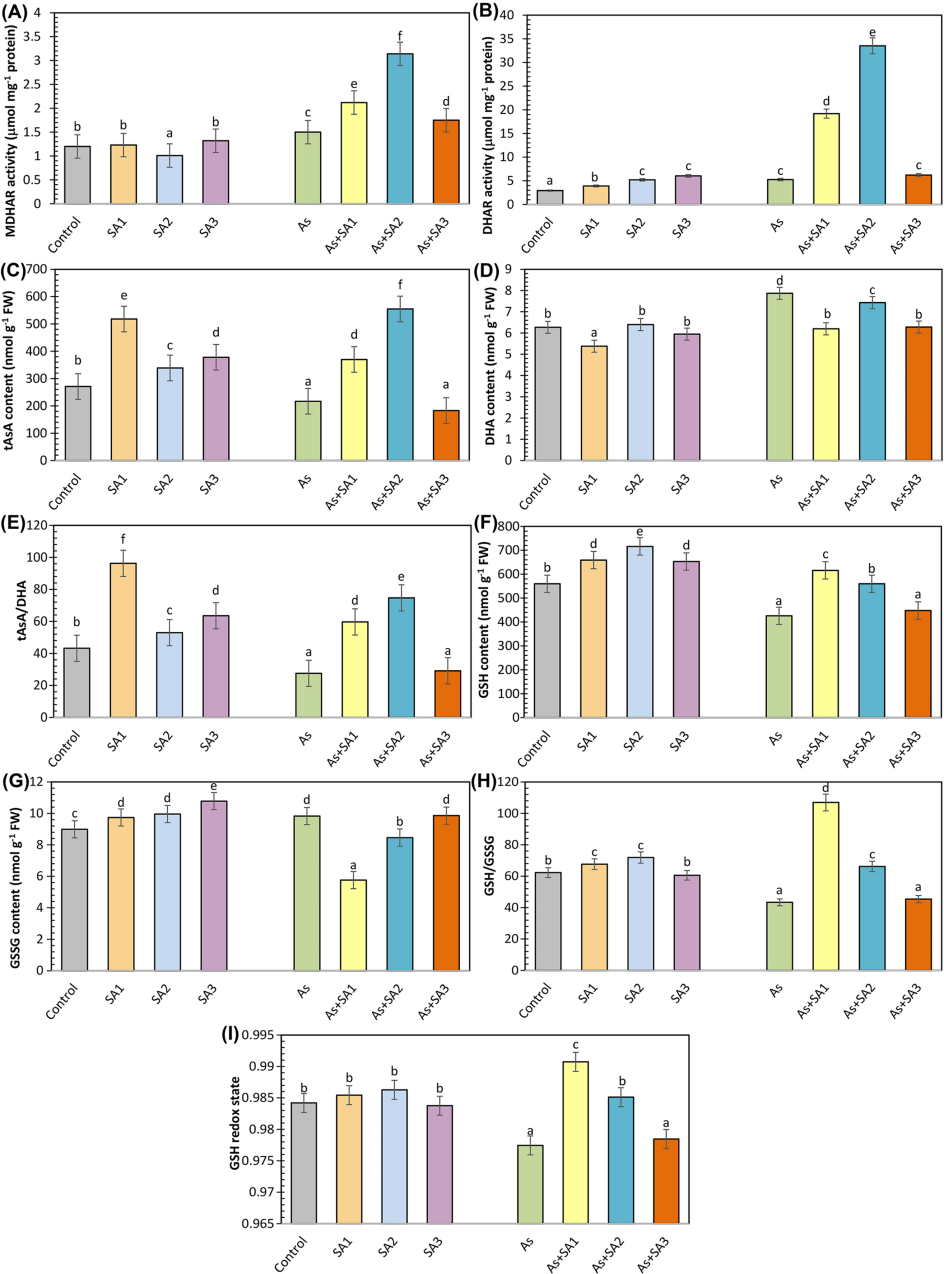
Effect of syringic acid treatment on ascorbate‐glutathione cycle and redox state in lettuce leaves under arsenic stress. The monodehydroascorbate reductase activity (MDHAR, A), dehydroascorbate reductase activity (DHAR, B), ascorbate content (AsA, C), dehydroascorbate content (DHA, D), tAsA/DHA (E), glutathione content (GSH, F), oxidized glutathione content (GSSG, G), GSH/GSSG (H) and GSH redox state (I) in syringic acid (SA1, 10 μM; SA2, 250 μM; SA3, 500 μM) treated lettuce leaves under arsenic stress (As, 100 μM; n = 6).

Under arsenic stress, MDHAR activity improved by 25% compared to the control, and simultaneous treatment with SA further enhanced this effect, especially in the As+SA2 group, where a 2‐fold growth was determined, and 41.3% in the As+SA1 group and 16.3% in the As+SA3 group, respectively (Figure 7A). DHAR activity increased by 33.1%, 77.4% and 2‐fold with SA1, SA2 and SA3 treatment, respectively, compared with the control (Figure 7B). Arsenic stress elevated DHAR activity by 79.5%, and co‐treatment with SA led to dramatic increases, especially in the As+SA1 and As+SA2 groups, where of 3.6‐fold and 6.3‐fold, respectively, were observed. SA treatments led to an induce in total ascorbate (tAsA) content against the control (Figure 7C). The SA1 treatment exhibited the greatest increase at 91.1%. The As group decreased the tAsA content by 19.9%. However, SA treatment improved tAsA content by 70% in the As+SA1 group and 2.5‐ fold in the As+SA2 group relative to arsenic stress. No remarkable difference was detected in the As+SA3 group. SA treatment did not alter DHA activity compared to control conditions, except in the SA1 group (Figure 7D). In addition, there was a 25% induction in DHA activity in the As group relative to control conditions. However, SA treatments decreased DHA activity in the stress group. As shown in Figure 7E, only SA treatments increased tAsA/DHA. In lettuce leaves subject to As stress, a 36.2% decrease in the tAsA/DHA ratio was detected in comparison with the control. This reduction resulted in an enhancement in the As+SA1 and As+SA2 groups. SA1, SA2 and SA3 treatments increased GSH content by 17.6%, 27.8% and 16.6%, respectively, as opposed to the control (Figure 7F). There was a 23.9% diminution in GSH content in the As group compared to control conditions. Additionally, SA treatments either did not show any change or a decrease was detected in GSH content relative to stressed plants. GSSG content was induced by SA1, SA2 and SA3 treatment compared with control (Figure 7G). Arsenic stress improved the GSSG content by 9.3%. SA treatments decreased the GSSG content in As+SA1 and As+SA2 groups, while no important distinction was recorded in As+SA3 group. We showed in Figure 7H, only SA treatments increased GSH/GSSG, except the SA3 group. Compared to the control, arsenic stress diminished the GSH/GSSG ratio by 30.4%. While As+SA1 and As+SA2 groups increased the GSH/GSSG ratio, a significant difference was observed in the As+SA3 group. Arsenic stress resulted in a reduction in the GSH redox level of lettuce plants (Figure 7I). Additionally, in plants treated with SA, either no change or an increase in GSG redox levels was observed relative to the stress group.

## 4 DISCUSSION

Arsenic toxicity has serious harmful impacts on plant growth, both inhibiting their development and causing significant decreases in crop productivity (Ahmed et al., 2024). In the present study, Arsenic stress led to a decrease in the RGR, RWC and Ψπ of lettuce seedlings. This decrease may be due to damage to ion channels in the cell membrane or closure of stomata (Kaur et al., 2024). In addition, the stress may be associated with a reduction in root hairs, leading to limited water and nutrient transfer (Ahmad et al., 2018; Sadeghipour and Monem, 2021). Maghsoudi et al., (2020) stated that the growth retardation under As stress was due to the suppression of photosynthesis rate and chlorophyll content. Consistent with our study, Zaheer et al., (2024) reported that As stress adversely impacted RGR and RWC of wheat seedlings. Additionally, Vezza et al., (2018) observed a notable decline in Ψπ and RWC of soybean leaves under As stress. Conversely, phenolic acids play a key role in promoting plant growth and increasing resistance to environmental stresses (Kaurinovic and Vastag, da‐Silva et al. 2019). In our study, as a result of treatments with different doses of SA, one of the phenolic acids, SA1 and SA2 treatments strengthened both growth and water content and osmoregulation in lettuce plants, but the SA3 dose was excessive and caused a toxic effect on the plants and negatively affected growth. The toxic effect of the SA3 dose may be associated with abnormalities in the nucleus, cytoplasm and vacuoles of root tip cells as the concentration of phenolic acids increases (Ma et al., 2024). Zhou et al., (2014) reported that SA‐treated cucumber seedlings also had a negative effect on growth. Considering this positive effect of SA1 and SA2, it has been suggested that phenolic compounds may increase cell division and elongation by stimulating the expression of genes associated with cell wall remodeling Ramzan et al., (2024). Wael et al., (2015) found that exogenous salicylic acid treated *Phaseolus vulgaris* plants under cadmium (Cd) stress increased growth and RWC by reducing the adverse impacts of Cd. Parvin et al., (2019) stated that quercetin treatment, which is among the phenolic compounds, increased the growth rate and alleviated RWC decreases under salt stress. In addition, Yildiztugay et al., (2019) stated that ferulic acid treatment increased the growth rate of wheat under boron stress and alleviated the decreases in RWC and Ψ_Π_. These studies support our findings. Proline functions as an osmotic protector by regulating ROS (Anas et al., 2024). Research has determined proline accumulation is frequently seen in plants under arsenic stress (Khan et al., 2021). In addition, as stated in many studies, increases in proline levels in stressed plants are considered a positive response of the plant's defense mechanism (Spormann et al., 2023). In our study, proline accumulation was induced in lettuce leaves under As stress. Alsahli et al., (2021) recorded similar results in pea plants under As stress. However, all SA treatments had reduced proline accumulation. This situation can be evaluated as phenolic acid treatment can be effective in water balance, as stated by Rahman et al., (2022). Likewise, Wang et al., (2013) found that ryegrass treated with salicylic acid reduced proline accumulation under Cd stress.

Heavy metal toxicity negatively affects *E*, g_s_ and C_i_ in plants (Zhou et al., 2024a). In this study, As stress negatively affected not only C_i_, *E* and A_c_ but also maximum quantum yield of photosystem II (F_v_/F_m_), and quantum yield of PSII photochemistry (F_v_/F_o_) values in lettuce leaves, with the decrease of g_s_. This condition may be associated with abnormalities in the stomata (Anjum et al., 2017). Moreover, it was documented that the functionality of PSII is compromised, and the deterioration of electron transport from PSII to PSI has damaged the photosynthesis mechanism (Nazir et al., 2023). Consistent with the findings of our study, Niazi et al., (2017) in *Brassica napus* and *Brassica juncea* plants, and Ghorbani et al., (2021) in tomato plants, stated that As stress caused impairments in photosynthesis and gas exchange parameters. SA1 and SA2 treated lettuce plants improved these parameters, resulting in increased photosynthetic efficiency, while SA2 showed less positive effects than SA1. This can be interpreted as exogenously treated SA1 and SA2 potentially increasing photosynthetic activity by helping F_v_/F_m_ and F_v_/F_o_ values return to the values observed in the control group. In addition, Saheri et al., (2020) stated that rubisco activity increases carboxylation efficiency by increasing carboxylation activity and correcting stomatal conductance, which decreases under stress conditions, thus making CO_2_ use more efficient and promoting growth under stress conditions. In this context, the research carried out by Colak et al., (2021) concluded that exogenous gallic acid application to wheat plants increased the F_v_/F_m_ value by improving the negative effects of stress, confirming our findings. On the other hand, SA3 treatment did not show any ameliorating or compensating effect against arsenic stress on F_v_/F_m_ and F_v_/F_o_ values. This shows that the SA3 treatment does not have a positive effect on the photosynthetic capacity and energy conversion efficiency of the plants. In parallel with our study, Lu et al., (2018) investigated the effects of exogenous SA on photosynthesis in strawberry seedlings and found that SA applied at different concentrations had an opposite effect by reducing F_v_/F_m_, g_s_ and C_i_ values in plants. In addition, Xie et al., (2018) stated in their study that with increasing phenolic acid concentrations, net photosynthetic rate, transpiration rate, chlorophyll content and total biomass decreased significantly, concluding that the higher the phenolic acid concentration, the more photosynthesis was inhibited. Exogenous SA1 treatment significantly improved photosynthesis, whereas SA2 provided milder benefits and SA3 did not reduce the adverse effects of As exposure. In our study, we also examined the JIP test data to evaluate the effect of As and SA on PSII efficiency and performance in more detail. PI_total_, one of the most important parameters of this test has been stated in studies as the most responsive and insightful photosynthetic parameter reflecting the activity of photosystems and the intersystem electron transport chain (Linić et al., 2021). In the present study, arsenic stress negatively affected the PI_total_, and PI_ABS_. But SA1 and SA2 treatment provided protection in lettuce plants by increasing the PI_ABS_ and PI_total_ values. Additionally, the stress‐relieving impact of SA on the ABS/RC, DIo/RC, TRo/RC parameters was evident, indicating that SA mitigated stress‐induced energy losses in the photosynthetic apparatus. This situation was reported by Elbasan et al., (2024), who reported results compatible with our study data, and they associated this with the smooth transmission of electrons between PSI and PSII, which led to a decrease in energy losses in the electron transport chain.

Many studies have shown that arsenic triggers ROS production (Nahar et al., 2022). Harmful ROS production negatively impacts the phospholipid bilayer, causing lipid peroxidation and a boost in TBARS content, which is an indicator of oxidative stress (Nabi et al., 2022). In the present study, arsenic induced TBARS content in lettuce plants. This can be attributed to the increased production of H_2_O_2_, consistent with our results. Our findings are also consistent with studies reporting that As causes significant lipid peroxidation in maize (Waheed et al., 2024), and *Brassica napus* (Bano et al., 2022). However, the SA3 treatment did not mitigate the adverse effects of stress. This may be related to the increase in H₂O₂ levels in the environment along with the increase in SOD activity and the insufficient activity of APX and CAT enzymes to remove this increased H₂O₂ from the environment. However, it was determined that SA1 and SA2 treatments suppressed the induced H_2_O_2_ and TBARS content, and SA1 treatment had a stronger effect on TBARS content. This means that SA protects against the breakdown of membrane lipids and the formation of harmful carbonyl compounds (Shahzad et al., 2020). Avdatek et al., (2023) reported that SA is a strong electron donor due to its phenolic structure regarding lipid peroxidation and the decrease in H_2_O_2_ levels and that it neutralizes ROS by converting them into stable, non‐toxic compounds. It has also been linked to the direct activation of antioxidant enzymes by the exogenous application of phenolic acids (Singh et al., 2017). Similar results have been observed by Moustafa‐Farag et al., (2020) in consequence of salicylic acid treatment of watermelon plants under boron stress. Additionally, Saidi et al., (2021) reported the results of gallic acid treatment on sunflower plants subjected to Cd stress. In the present study, all SA treatments, especially SA1, increased SOD activity. Moreover, SA1 and SA2 treatments substantially increased POX, APX and GPX activities. The enhanced activities of these enzymatic antioxidants may be attributed to the free radical scavenging capacity of SA (Kumar et al., 2012). According to our investigations, SOD, which converts superoxide anions to H_2_O_2_, and POX, APX and GPX enzymes involved in the clearance of H_2_O_2_, provided effective protection. Similar findings were seen in the study of El‐Soud et al., (2013) who reported that the treatment of ellagic acid to chickpea seedlings under osmotic stress caused an increase in POX, SOD, APX, and GR enzymes. Additionally, they demonstrated that the increased antioxidant enzyme activities were linked to the upregulation of the corresponding enzyme genes (Yin et al., 2024). In other words, it was reported that under environmental stress conditions, phenolic acids regulate antioxidant response pathways and enhance enzyme expression by activating the associated enzyme genes (Liu et al., 2024). Osei et al., (2022) stated that salicylic acid application in stressed potato plants strengthened the antioxidant defense system by increasing the expression of SOD and POX genes. Xuan and Khang (2018) reported that vanillic acid application to rice plants under water stress increased the expression level of APX genes. In our study, a significant induction in NOX, which contributes to ROS production, was also observed. Lin et al., (2021) indicated that some citrus genes encoding NOXs in orange induce the accumulation of ROS during the initial plant defense responses in infected areas. In addition, GST enzyme activity, which is engaged in the detoxification mechanism of plants, also increased in SA applications alone and in the As+SA1 group. Sultana et al., (2024) proposed that a higher GST level is crucial for safeguarding plants from toxic aldehydes. These results are supported by the study by Singh and Roychoudhury (2023). CAT activity did not show a significant change in the As+SA1 group as a result of SA application together with As, but diminished in the As+SA2 and As+SA3 groups. Antonić et al., (2020) obtained similar results in their study measuring the drought response of salicylic acid applications in *Impatiens walleriana* and stated that salicylic acid can inhibit the CAT enzyme by direct binding. Additionally, in the present study, GR enzyme activity was induced in the As+SA1 and As+SA2 groups and suppressed in the As+SA3 group. Increased GR activity has been associated with a higher GSH/GSSG ratio (Ahmad et al., 2011). Ma et al., (2024) stated that phenolic acids may have different effects at low and high concentrations, while high concentrations may cause a general weakening of enzymatic activity. In our research, an increase in MDHAR activity was observed in As+SA1, As+SA2 and As+SA3 groups, while an increase in DHAR activity was detected in As+SA1 and As+SA2 groups. This may be due to its critical role in maintaining higher substrate concentrations for APX (Namdjoyan et al., 2017). In addition, the fact that the As+SA3 group did not show any change under stress may be because the DHAR enzyme did not work at this concentration. SA1 and SA2 treatments with stress enhanced AsA and GSH contents and declined DHA and GSSG contents. Thus, SA1 and SA2 helped reduce oxidative damage in lettuce plants under As stress by increasing both AsA/DHA and GSH/GSSG ratios. Shahzad et al., (2020) stated that SA limits the severity of oxidative stress along with increasing GSH levels. Similar findings were detected in tomato plants treated with vanillic acid (Parvin et al., 2020). On the other hand, SA3 treatment with arsenic stress did not show any significant change on the negative effects of stress on AsA and GSH contents. Bashri and Prasad (2016) stated that low doses of exogenous indole‐3‐acetic acid application reduced toxicity by stimulating the AsA‐GSH cycle in plants under Cd stress, while high doses had the opposite effect, suppressing DHAR and GR enzymes and impairing antioxidant capacity. In line with these results, SA3 treatment had a toxic effect, especially on plant growth and most of the other parameters (photosynthesis, H_2_O_2_ content, TBARS content, POX, GST, GPX, DHAR activity, tAsA, GSH and GSSG content), indicating that this dose is not suitable for lettuce plants. Although SA2 treatment also showed positive effects on plants, it was insufficient compared to SA1. In our study, the most pronounced remedial effect among the different SA doses was observed with the dose designated as SA1 under arsenic stress conditions. SA1, determined as the appropriate dose, played a crucial role in the adaptation of lettuce plants to arsenic stress conditions by showing the most significant positive effect on both growth parameters and physiological stress indicators and enzyme activities.

## 5 CONCLUSION

In this study, the impacts of different doses of SA treatments on lettuce plants under arsenic stress conditions were comprehensively investigated. Our study covers a multitude of physiological and biochemical processes like plant growth performance, gas exchange parameters, photosynthetic parameters, ROS content, lipid peroxidation levels and antioxidant enzyme activities. In this direction, it was determined that As stress diminished RGR, RWC and Ψ_Π_ in lettuce plants and physiological processes were negatively affected. Additionally, arsenic stress caused suppression of gas exchange parameters (A_c_, C_i_ and *E*) and photosynthetic efficiency (F_v_/F_m_, F_v_/F_o_) in lettuce plants. SA1 and SA2 treatments improved the photosynthetic performance, growth and water relations of plants by alleviating the negative effects. Additionally, these treatments increased the resistance of plants to As‐induced toxicity by reducing H_2_O_2_ and TBARS levels, which are key indicators of oxidative stress. However, SA1 treatment was found to have a stronger effect on these parameters compared to SA2. In addition, SA1 and SA2 treatments induced antioxidant defense systems such as SOD, POX, GPX, APX, GR, MDHAR, DHAR, tAsA and GSH, consistent with decreasing H_2_O_2_ levels. On the other hand, SA3 treatment negatively affected plant growth. Although it increased SOD and MDHAR activities, it did not show any significant change in alleviating the negative effect of arsenic stress on parameters such as photosynthesis, oxidative stress markers, antioxidant enzyme activity, and redox regulation. In conclusion, SA1 treatment demonstrated its effectiveness in reducing arsenic stress. Furthermore, the results indicate that SA1 treatment plays a critical role in preventing yield losses by gaining resistance in plants under arsenic stress and offers new possibilities in agricultural production.

### Author Contributions Statement

E.Y., C.O.K. and B.A.A. conceived and designed research. B.A.A. and M.B. conducted the experiments. E.Y., B.A.A., C.O.K., and M.B. analyzed the data. M.B., B.A.A., and E.Y. wrote the manuscript. All authors read and approved the manuscript.

## Supporting information


**Supplementary Table S1.** Parameters derived from the portable fluorometer and OJIP transient and their definitions for use in the current study.

## Data Availability

Data sharing is not applicable to this article as all new created data are already contained within this article.
