# Supplementary material for: Role of syringic acid in enhancing growth, photosynthesis, and antioxidant defense in lettuce exposed to arsenic stress
Source: Physiol Plant. 2025 Jan 15;177(1):e70051. doi: 10.1111/ppl.70051 (PMC11744429; doi:10.1111/ppl.70051)
Supplement: Supplementary file 1 — Supplementary Table S1. Parameters derived from the portable fluorometer and OJIP transient and their definitions for use in the current study. [file PPL-177-e70051-s001.docx]

| **OJIP-Derived Parameter** | **Definition** |
| --- | --- |
| **Chlorophyll fluorescence and gas exchange** | |
| F_v_/F_m_ | The maximal quantum yield of PSII photochemistry |
| F_v_/F_o_ | The efficiency of donation electron to PSI |
| F_o_/F_m_ | The physiological state of the photosynthetic apparatus |
| **Quantum Efficiencies** | |
| ΨE_o_ | Quantum yield of electron transport to intersystem, the efficiency that an electron moves further than Q_A_. |
| ϕR_o_ | Quantum yield for reduction of the PSI end electron acceptors: Ferredoxin and NADP. |
| **Structural Indicators and Fluxes** | |
| ABS/RC | Ratio of antenna chlorophyll to PSII reaction center chlorophyll (indicator of antenna size for PSII). |
| TR_o_/RC | Energy trapping per reaction center: reduction of Pheophytin and Q_A_. |
| ET_o_/RC | Electron transport per reaction center: beyond Q_A_^−^. |
| DI_o_/RC | Dissipation flux per active reaction center |
| dV/dt_o_ | Q_A_ maximum reduction rate |
| ΦP_o_/(1-ΦP_o_) | Q_A_-reducing reaction centers per PSII antenna chlorophyll |
| ΨE_o_/(1-ΨE_o_) | The efficiency with which a trapped exciton transfers an electron to the photosynthetic electron transfer chain |
| γRC/(1-γRC) | The fraction of PSII chlorophyll a molecule that function as reaction centers |
| V_J_ | Relative variable fluorescence at the J- (2 ms) |
| V_I_ | Relative variable fluorescence at the I- (30 ms) |
| **Performance Indices** | |
| PI_ABS_ | Multi-parameter photosynthetic performance index expressing energy conservation from absorption of light by antenna complexes of photosystem II to electron transport to intersystem electron acceptors. |
| PI_total_ | Multi-parameter performance index expressing energy conservation from absorption of light by photosystem II electron acceptors to PSI end electron acceptor reduction. |

**Supplementary Table S1.** Parameters derived from the portable fluorometer and OJIP transient and their definitions for use in the current study
